# Epigenetic marks as the link between environment and development: examination of the associations between attachment, socioeconomic status, and methylation of the *SLC6A4* gene

**DOI:** 10.1002/brb3.480

**Published:** 2016-06-09

**Authors:** Karen Jones‐Mason, Isabel Elaine Allen, Nicole Bush, Steve Hamilton

**Affiliations:** ^1^ Department of Social WelfareUniversity of California, BerkeleyBerkeleyCalifornia; ^2^ Department of StatisticsUniversity of California, San FranciscoSan FranciscoCalifornia; ^3^ Department of Psychiatry and PediatricsUniversity of California, San FranciscoSan FranciscoCalifornia; ^4^ Department of PsychiatryUniversity of California, San FranciscoSan FranciscoCalifornia; ^5^ Department of PsychiatryKaiser‐Permanente San Francisco Medical CenterSan FranciscoCalifornia; ^6^ Center for Health & CommunityUniversity of CaliforniaSan FranciscoCalifornia

**Keywords:** Attachment, socioeconomic status, methylation, *SLC6A4* and 5‐HTTLPR

## Abstract

**Background:**

Epigenetic processes act as a link between environment and individual development. This pilot study examined the association between socioeconomic status (SES), attachment, and methylation of the promoter region of the serotonin transporter gene (*SLC6A4*).

**Methods:**

Attachment classification and *SLC6A4* methylation was determined in 100 late adolescents. We hypothesized that (1) SES would interact with methylation to predict higher unresolved loss (UL) or trauma scores on the Adult Attachment Interview; (2) across SES, participants with unresolved attachment would have lower levels of methylation than organized or secure participants; and (3) within the unresolved classification, SES would predict methylation.

**Results:**

Results showed that lower methylation and low‐SES were associated with higher UL, and higher methylation and low‐SES were associated with higher unresolved trauma. Across SES, unresolved participants had lower levels of* *methylation than organized participants. Within the unresolved category, low‐SES unresolved participants had higher levels of methylation than mid/upper‐SES participants. SES was unrelated to methylation within the secure and organized categories.

**Conclusions:**

These results suggest that the quality of attachment relationships may impact epigenetic processes.

## Introduction

Development is the result of complex interactions between genetic, environmental, and other biological factors (Hernandez and Blazer 2006; McDade et al. 2006; Rutter 2006; Danese et al. 2007). Current research suggests that genes can be activated or silenced in response to environmental signals, a process that can be triggered by a broad range of events including exposure to pollutants, medications, diet, and social experience (Sweatt et al. 2013; Tammen et al. 2013). Some of the earliest signals the human genome receives come from the infant–caregiver attachment relationship. The attachment relationship is important not only because it provides the first critical developmental environment humans encounter, but also because signals received within the attachment context come at a time when the brain is particularly plastic (Graham et al. 2013; Sale et al. 2014). In addition, evidence suggests that attachment relationships play a role in stress regulation and health outcomes throughout the life span (McWilliams and Bailey 2010; Nolte et al. 2011; Puig et al. 2013). Although the importance of attachment relationships is widely recognized, little is known about the associations between attachment, socioeconomic status (SES), and human DNA methylation. This study explored whether attachment organization may act as a protective factor against the negative health outcomes associated with low SES. Specifically, the study examined associations between attachment classification as assessed by the Adult Attachment Interview (AAI), and methylation of the serotonin transporter gene (*SLC6A4*), and whether SES modifies these associations.

### Genes and environment

Researchers over the last decade have advanced understanding of the connection between genes, environmental stress, human development, and health (Kochanska et al. 2009; Ellis et al. 2011; Mitchell et al. 2014). In particular, the *SLC6A4* gene, which plays a critical role in brain development and emotion regulation (Lesch 2007; Booij et al. 2013), has been extensively studied—especially the 5‐HTTLPR polymorphic region of the promoter characterized by the presence of a short “s” allele or a long “l” allele (Caspi and Moffit 2006; Taylor et al. 2006; Kogan et al. 2010). Possession of at least one s allele (ss and sl genotypes) has been associated with reduced transcriptional efficiency (Barry et al. 2008), a smaller amygdala and cingulate cortex, and weaker signaling between those brain regions, relative to ll individuals (Pezawas et al. 2005). Because carriers of the s allele may have more trouble reducing amygdala activation, some researchers have suggested that individuals who have the s allele are at higher risk of unresolved loss (UL) or trauma (UT) (Caspers et al. 2009).

Caspi et al.'s (2003) finding that stressful events in adulthood predicted more depressive symptoms for individuals who carried the s allele of *SLC6A4* compared to those who possessed the homozygous longer variant (ll genotype) provoked a great deal of research examining whether *SLC6A4* genotype predicts sensitivity to the environment. A recent meta‐analysis (van IJzendoorn et al. 2012) concluded that s‐carriers were more sensitive to positive *or* negative environmental experience than ll‐carriers. Findings also suggest that this genetic sensitivity to experience includes interactions with family members (Taylor et al. 2006; Kochanska et al. 2009; Ellis et al. 2011; Mitchell et al. 2014). For example, Taylor et al. found that adults with the ss genotype from supportive family environments had the lowest depressive symptomology, whereas those who experienced a stressful early life environment had the highest level of depressive symptoms. Moreover, animal and human studies also suggest that parenting behaviors may influence serotonergic functioning (Francis et al. 1999a,b; Ichise et al. 2006; Caspi and Moffit 2006; Shannon et al. 2006; Taylor et al. 2006; Kinnally et al. 2008; Beach et al. 2010, 2015), pointing to the importance for investigating how family relationships might affect serotonin gene regulation.

The last two decades, however, have been marked by conflicting findings from research examining the association between adverse life experiences, psychopathology, and the *SLC6A4* gene, as well as debate regarding overall methodology for G × E investigations (see, for example, Duncan and Keller 2011). Although two meta‐analyses concluded that there was no significant association between the *SLC6A4* genotypes and psychopathology (Munafò et al. 2009; Risch et al. 2009), more recent reviews have concluded otherwise. Uher and McGuffin (2008, 2010) argue that those studies which failed to find a G × E interaction used self‐report measures to assess environmental adversity, whereas those studies that used contextual or objective measures, including semi‐structured interviews, confirmed G × E findings. Inconsistent findings in genotype × environment studies may not only be attributable to factors such as the use of self‐report measures, but may also result from a lack of consideration of factors such as variation in gene regulation. This study addresses these issues by using the AAI instead of a self‐report measure of attachment, and examining the association between attachment classification and epigenetic marks.

### Epigenetics

Epigenetics is the study of the way the environment regulates gene activation (Boyce and Kobor 2015). *Epigenetic* literally means “above genetics” and refers to genetic change that does not involve the nucleotide sequence (Allis et al. 2007). In general, epigenetic processes provide ways for cells to specialize and adapt to environment. During methylation, the most widely studied epigenetic process (Umer and Herceg 2013), enzymes attach methyl groups to regions of DNA referred to as CpG islands. DNA methylation is typically associated with gene silencing, and is considered to be the most stable epigenetic mark (Booij, Wang, Levesque, Tremblay & Szyf, 2013). For these reasons, methylation processes may serve as an interface between the neurobiological basis of development and environmental contexts (Ellis et al. 2011). Indeed, findings from the recent explosion of related animal research suggest that early life experiences with parents impact the development of offspring through epigenetic processes such as methylation (Fish et al. 2004; Meaney and Szyf 2005). Today, multiple lines of research suggest that parental sensitivity to a child's signals for protection, comfort, or assistance helps to regulate the child's emotional reactivity to environmental stimuli and thus impacts both neurological structure and gene regulation, particularly those genes and parts of the brain related to stress regulation (Murgatroyd and Spengler 2011; Bock et al. 2014; Szyf and Bick 2014; Beach et al. 2015).

### *SLC6A4* and attachment

The relation between genotype and attachment classification remains a complex area of research, with some studies finding an association between the ss genotype (or s allele) and adult or infant attachment classification (Caspers et al. 2009; Spangler et al. 2009; van IJzendoorn et al. 2010), and others failing to find such a relationship, or showing conflicting or mixed results (Luijk et al. 2011; Raby et al. 2012, 2013). A recent study, using data from the NICHD Study of Early Child Care and Youth Development, with a sample of over 600, examined genetic associations with infant attachment and concluded that the effect of various dopaminergic, oxytonergic, and serotonergic polymorphisms on attachment was essentially negligible (Roisman et al. 2013). The reasons for the inconsistent and weak results are likely multifactorial, but may also include a failure to take into account epigenetic processes. In other words, it may not be genes per se that are important for understanding the relationship between attachment and biology, but rather gene regulation.

### Epigenetic marks within *SLC6A4* and attachment

Few studies have examined the association between *SLC6A4* methylation and attachment. Several extant studies were conducted by Philibert and colleagues using the Iowa Adoption Study (Philibert et al. 2007; Beach et al. 2011; van IJzendoorn et al. 2010). This group reported that abuse experienced in childhood, including physical and sexual abuse, was correlated with hypermethylated *SLC6A4* upstream CpG islands in females (Beach et al. 2010). Methylation levels also correlated with a history of childhood sexual abuse and with symptoms of Antisocial Personality Disorder in female subjects and appeared to potentiate the influence of the short genotype of the 5‐HTTLPR polymorphism (Beach et al. 2011). Kang et al. (2013) found that increased *SLC6A4* methylation was associated with higher levels of childhood adversity, stress, psychopathology and a family history of depression. In the only other study we know that has examined the associations investigated in the present research, van IJzendoorn et al. (2010) found that lower levels of *SLC6A4* methylation in participants homozygous for the short genotype of 5‐HTTLPR predicted increased risk of UL and trauma as coded by the AAI, in a sample of primarily Caucasian middle‐class adults adopted as infants.

### Socioeconomic status, attachment and epigenetics

Low‐SES may influence methylation (Borghol et al. 2011; Tehranifar et al. 2013; Beach et al. 2014b), and it is associated with a higher risk of insecure and unresolved attachment classification (van IJzendoorn and Bakermans‐Kranenburg 1996). Accordingly, consideration of the potential contribution of SES to the associations reviewed above is merited.

Generally, low‐SES is thought to impact health, including stress‐related diseases, through environmental experiences that influence gene regulation (Miller et al. 2009). Attachment classification itself may also be related to health and stress‐related diseases (McWilliams and Bailey 2010), the mediator for which may be epigenetic processes such as methylation. For example, in a low‐SES sample, Puig et al. (2013) found that adults who were classified as secure in infancy reported lower levels of disease in adulthood than those who were classified as insecure. Brody et al. (2013) found that in a low‐SES sample of African‐American youth, among those youth who carried two genes for environmental sensitivity (5‐HTTLPR s allele and *DRD4 7* + R allele), those who grew up in a “supportive family environment” had a lower “allostatic load” than those youth who grew up in an “unsupportive family environment.” Chen et al. (2011) found that participants who were raised in low‐SES homes who reported high levels of maternal warmth showed lower levels of inflammation‐related gene expression compared to those who reported low levels of maternal warmth. Although maternal warmth and family support are not the same constructs as attachment, these studies point to the possibility that attachment security could provide some buffer against the detrimental impact of a low‐SES environment.

### This study

The overall aim of this study was to examine the associations between methylation, SES and unresolved attachment. The term “unresolved loss or trauma” (hereinafter ULT) refers to the failure of an individual to fully “integrate” into conscious awareness the loss of an attachment figure or a traumatic experience (Lyons‐Ruth et al. 2003). Because not everyone who loses a loved one becomes unresolved, it is reasonable to ask whether individual differences in biology, such as epigenetic processes, may make some individuals more vulnerable to unresolved attachment. On the other hand, epigenetic methylation may also be a response to specific social events such as trauma or low‐SES. Accordingly, for this study we examined unresolved loss (hereinafter UL) and unresolved trauma (hereinafter UT) effects separately. Following previous studies (Caspers et al. 2009), we used both the continuous and categorical scoring produced by the AAI in our analyses, which increases confidence in results. In addition, because genotype may affect methylation (Beach et al. 2014a,b), and because previous studies identify an association between methylation, genotype and attachment (van IJzendoorn et al. 2010), we also controlled for genotype as appropriate within models. The study had four main hypotheses; the first three use continuous data (ULT, UL, UT) and the fourth uses categorical data (secure, organized (insecure plus secure) and unresolved).

1. Unresolved loss and trauma (ULT): First, we examined the main effects of SES and methylation, and their interaction, in association with ULT. We hypothesized, that, based on well‐established literature, low‐SES would be associated with higher levels of ULT. However, previous studies have found both that *higher* levels of *SLC6A4* methylation were associated with trauma experiences (Beach et al. 2010; Vijayendran et al. 2012), and that *lower* levels of *SLC6A4* methylation were associated with ULT (van IJzendoorn et al. 2010). Accordingly, we did not have a specific directional hypothesis for this first analysis.
2. Unresolved Trauma (UT): We hypothesized that, based on previous findings, low‐SES and *higher* levels methylation would be associated with higher UT.
3. Unresolved loss (UL): We hypothesized that low‐SES and *lower* levels of methylation would be associated with higher UL. Note that we could not base these hypotheses on van IJzendoorn et al. (2010) findings alone since that study involved solely ULT, but we reasoned that it would make sense for individuals with low *SLC6A4* methylation, and therefore an activated “sensitivity” gene, to be at greater risk for UL; such a pattern would account for the conflicting patterns of associations found between the Beach et al. (2010) and Vijayendran et al. (2012) studies and those from van IJzendoorn's group. Also recall that van IJzendoorn et al. (2010) involved a low‐risk sample, suggesting that the study probably had more UL than trauma. Because of concerns that trauma experiences may impact methylation for participants with concurrent loss and trauma, reducing our ability to identify the effect of loss, we also conducted additional analyses involving only participants with loss but no trauma.
4. Attachment as a categorical variable: We next sought to understand whether we could identify an association between categorical attachment classification, SES and *SLC6A4* methylation.
  - Across SES: In line with related findings from van IJzendoorn et al. (2010), we hypothesized that across SES, participants classified as categorically unresolved would have lower levels of *SLC6A4* methylation than secure participants.
  - Incorporating SES: Given previous research suggesting that social experience such as low‐SES may impact *SLC6A4* methylation (Beach et al. 2014a,b) we expected that low SES participants would have higher levels of methylation than mid/upper SES participants. Moreover, we expected that the relationship between methylation and attachment classification would change once we considered SES; lower SES participants classified as unresolved would have *higher* levels of *SLC6A4* methylation than mid/upper SES unresolved participants. Based on previous research suggesting secure attachment may have protective health benefits (Puig et al. 2013), and given the association of disorganization with high risk/low SES environments, we expected that SES would not be related to *SLC6A4* methylation among secure or organized participants.
  - Comparing categorical and continuous analyses. Finally, to facilitate a comparison with the analysis conducted with the continuous UL and trauma scores, we added a linear regression analysis to determine whether the significance of the relationship between attachment and methylation changed when attachment was made a dependent variable, while covarying for SES and methylation.

Overall, in addition to breaking out UL and UT, these analyses advance the work of van IJzendoorn et al. (2010) in at several respects: First, we incorporated SES into all our models and tested for interactions between SES and methylation. Second, participants were late adolescents who lived with at least one biological parent (in contrast to adults adopted as infants). Third, DNA was obtained from blood directly, not buccal cells as in Caspers et al. (2009) or transformed lymphoblast cell lines as in van IJzendoorn et al. (2010). Finally, to facilitate comparison with related existing literature, we also conducted a regression analysis attempting to replicate their results that showed that ss genotype and lower *SLC6A4* methylation predicted higher ULT.

## Research Design and Methodology

### Participants

The study population consisted of 101, primarily female (*n* = 82, 81.2%), late adolescents (mean age = 19.8 years) attending a large public university in the western United States. Methylation analysis was unsuccessful for one participant, reducing the analytic sample size to 100. Approximately 34% of the sample self‐identified as Euro‐American, 49% as Asian American, 12% as Hispanic, and 2% African‐American.

### Procedure

All procedures were approved by the University of California's Committee for the Protection of Human Participants (CPHS). Participants were recruited from a university web site and from campus fliers. Exclusionary criteria eliminated any potential participant who had used psychotropic medications or glucocorticoids within the last month. After giving written consent, students completed questionnaires. The AAI was administered thereafter by specially trained individuals. In approximately half the cases, a blood draw was made before the measures were administered, and in half the cases the blood draw was made afterward. All blood was taken at the university health center by licensed phlebotomists. All measures were administered in a private office to ensure complete confidentiality.

### Measures

Participants self‐reported ethnicity and sex. The widely used Hollingshead Measure of SES (HSES) (Hollingshead 1975) was used for assessing SES. The HSES creates five levels of SES ranging from “unskilled laborers” to “major business and professionals.” Following the approach taken by other studies, we created a dichotomous variable for analyses combining the bottom two levels to constitute “low‐SES” and the top three to comprise “mid/upper SES” (Yin et al. 2012).

The Beck Depression Inventory—II (BDI‐II) and the Beck Anxiety Inventory (BAI) were used to assess current levels of depression or anxiety (Beck et al. 1996). The BDI requires participants to rate 21 symptoms associated with depressed mood that may have occurred during the prior 2 weeks on a scale ranging from 0 (not present) to 3 (severe). Scores range from 0 to 63. Strong internal consistency and convergent validity has been reported (Beck et al. 1996). Similarly, the BAI (Beck et al. 1961, 1988) requires participants to answer 21 questions about symptoms of anxiety that they may have experienced in the last week (e.g., sweating, numbness, trembling, etc.). The BAI has been found to be internally consistent and reliable (Cronbach's α of 0.94 and test–retest reliability coefficient of 0.67) (Fydrich et al. 1992).

Attachment state of mind was measured through the use of the AAI. The AAI is a semi‐structured interview for adolescents/adults about childhood experiences with attachment figures and the meaning the individual gives to those experiences in the present (George et al. 1984/1985/1996). The AAI has demonstrated construct validity and reliability (George et al. 1984/1985/1996) as well as test–retest reliability (Sagi et al. 1994). During the AAI, the interviewee is asked to give a general description of their childhood relationship with primary caregivers. The interviewee is then asked to give five adjectives that describe their *relationship* with their attachment figures, as well as specific memories that support those adjectives. In addition, the interviewee is asked about experiences when they were hurt, frightened, or ill. Finally, the interviewee is asked about experiences of loss and abuse, the meaning that he or she attributes to all these experiences and how they apply to the interviewee in terms of his or her personality and own parenting. The interview is then transcribed and evaluated for what is called “coherence.” Main et al. (2003) adopted the following definition of coherence; “…a connection or congruity arising from some common principle or relationship; consistency; connectedness of thought such that the parts of the discourse are clearly related, form a logical whole or are suitable or suited and adapted to context” (p. 46). In other words, the crucial question is whether the interviewee is able to provide a believable and integrated (i.e., logical, relevant, concise but complete, and clear) account of experiences and their meaning. The transcript is then assigned to one of four classifications: “autonomous” (a secure category—designated the “F” category); two insecure categories—”dismissing” (an avoidant category—designated the “D” category), and “preoccupied” (an ambivalent/resistant category—designated the “E” category); and for interviewees who report attachment‐related traumas of loss and/or abuse, and who demonstrate confusion and disorganization during the interview, a fourth category called “unresolved” (designated “U”). Participants who are classified as unresolved also receive a secondary organized classification (i.e., F, D or E). A fifth classification is called “cannot classify” (designated “CC”) and refers to individuals who show the presence of multiple states of mind with respect to attachment. The CC classification is correlated with high risk of psychopathology (Crowell et al. 1999; Hesse 1999; Dozier et al. 2008), and is commonly grouped with participants falling in the U classification (Ward et al. 2006).

Participants may also be assigned four possible continuous unresolved scores associated with UT, UL or ULT (a score consisting of the highest loss or trauma score) and “other” trauma (e.g., trauma stemming from a car accident). As noted, the AAI assesses unresolved loss (hereinafter UL) and unresolved trauma (hereinafter UT) separately. The continuous scores use a scale of 1–9. Elevated scores (e.g., above 5) for UL or UT result in the assignment of a categorical classification referred to as “U” (unresolved). Accordingly, the AAI produces both a continuous score for UL and UT as well as a categorical classification (“U”). Note that ULT is simply the UL or UT score that is the highest. This study had no meaningful levels of “other” trauma so those scores are not included here.

Interviews were audiotaped and transcribed by an individual experienced in transcribing AAIs. Inter‐rater reliability between coders for the AAI was high for general classifications. All raters were certified as reliable by the Berkeley laboratory of Mary Main and Erik Hesse, the Jacobvitz, lab in Austin, Texas, or the Sroufe, lab in Minnesota. All raters were blind to each other's coding, and to any statistical data generated in the study. Inter‐rater reliability between rater 1 and rater 2 was made on the basis of 16 transcripts. Inter‐rater agreement scores across all four classifications were satisfactory (*K* = 0.77; 86.7% agreement). In addition, intra‐class correlation coefficients (ICC) between coders' rating scores for continuous UL (=0.90, *P* < 0.001) and UL or trauma (ICC = 0.91, *P* < 0.001) were positive and significant. All AAI coders were blind to methylation levels and the lab was blind to AAI coding.

#### Methylation measures

Gene methylation was measured by sodium bisulfate methylation mapping. DNA was obtained from peripheral lymphocytes using standard salting out methods. An assay was created for the Sequenom Mass Array system with the EpiTYPER assay. Samples were then treated with bisulfite to convert unmethylated cytosines to uracil. The regions of interest for *SLC6A4* were amplified using standard polymerase chain reaction (PCR) methods. Bisulfite‐treated DNA underwent in vitro RNA transcription, followed by a base‐specific cleavage reaction. This cleavage product leads to a mass difference for every methylated base that results in distinct signals when measured in a mass spectrometer (Zilberman and Henikoff 2007). Methylation results are reported in terms of percentage methylation. For example, a result of 0.03 means that 3% of sites were methylated in a particular CpG residue. The process is reported in greater detail in Zilberman and Henikoff (2007). Methylation analyses were performed at the UCSF Helen Diller Family Comprehensive Cancer Center Genome Analysis Core.

#### Genotype

PCR‐based genotyping methods were used for genotyping this repeat polymorphism (Kraft, et al., 2007). “Long” (16 repeat, 419 bp PCR fragment) and “short” (14 repeats, 376 bp PCR fragment) alleles were separated by electrophoresis and scored. Samples from five participants required a second attempt at genotyping (at the same lab) after initial testing failed to identify genotype. As has been done previously (Xie et al. 2012), a continuous variable for genotype was created based on number of “l” alleles: 0 (ss), 1 (sl) and 2 (ll).

**Figure 1:**
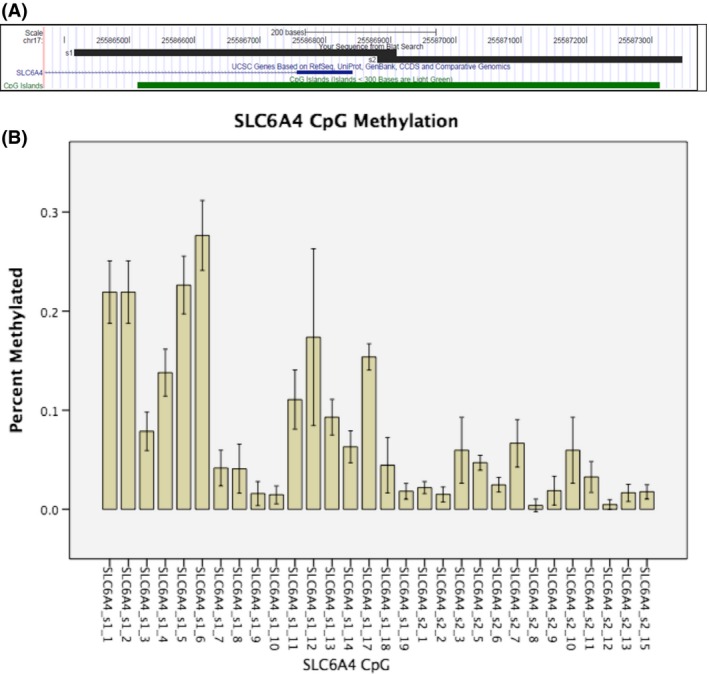
Targeted regions for methylation data, and mean *SLC6A4* methylation. (A) Targeted regions for methylation data. Two amplicons used to cover *SLC6A4* in CpG island upstream of exon 1. (B) Mean methylation, with standard deviation, for two *SLC6A4* amplicons for 100 participants.

This study targeted regions of the *SLC6A4* gene for methylation analysis that are commonly used in the research literature (McGowan et al. 2009; van IJzendoorn et al. 2010) (Fig. 1). We identified data for 34 CpG sites for *SLC6A4*. Thirty CpG sites met the requirement of >5% difference in methylation between minimum and maximum methylation fraction among the participants; the four sites that did not meet this requirement were not included in Fig. 1, but were included in the final analysis in order to adjust the findings. Figure 1 schematizes the targeted regions for the methylation analysis in the upper chart, and in the lower chart, mean levels of methylation are shown for 100 participants.

### Analytic models

All main models controlled for sex and ethnicity (Asian American vs. others). Ethnicity was included as a covariate because the only differences detected in genotype or in *SLC6A4*_1pc methylation between ethnic groups was between Asian Americans and Euro‐Americans (χ^2^ = 22.606, *P* < 0.001, *F *=* *8.031, *P *=* *0.006, respectively). Participants from remaining ethnic groups were added to the “other” category out of an abundance of caution. Other analyses conducted to avoid confounding are reported in Table A1 in Appendix.

Because of concerns that multiple testing will obscure results that are clinically relevant, principal component analysis (PCA), a technique that creates a weighted average of methylation levels for each participant, is commonly used in epigenetic studies (Lam et al. 2012). Analyses presented here used the same approach of creating a PCA of overall methylation across CpGs as described in Beach et al. (2010) and as described as a “weighted average” in van IJzendoorn et al. (2010).

After applying PCA to our methylation data, multiplicity was thereafter reduced from 34 CpG's to 2 sets (*SLC6A4*_1pc and *SLC6A4*_2pc). Accordingly, all testing for differences in methylation used the two principle components, *SLC6A4*_1pc and *SLC6A4*_2pc, for the *SLC6A4* gene.

Linear regression was used to examine the relationship between *SLC6A4* methylation and the continuous attachment scores (ULT, UL, and UT). Univariate ANOVA was used to conduct the analyses with the categorical attachment outcomes. Because our data were cross‐sectional, and it is also plausible that that social experiences such as attachment and SES impact methylation marks, we conducted the analyses using methylation as a dependent variable, and SES and attachment category as independent variables. Structuring the analysis in this manner also facilitated the ability to test for SES effects within each attachment classification. A linear regression analysis was added to determine whether the significance of the relationship between attachment and methylation changed when attachment was made a dependent variable while covarying for SES and methylation, and to facilitate a comparison with the analysis conducted with the continuous UL and trauma scores, also appears in this section.

## Results

Table 1 presents descriptive statistics. The distribution of AAI classifications (secure‐autonomous [F], insecure‐preoccupied [E], insecure‐dismissing [D], and unresolved [U]) was consistent with the reported rates in large surveys (van IJzendoorn 1995; Bakermans‐Kranenburg and van IJzendoorn 2009) (see Table 1). Approximately 47.5% of the sample was classified as secure, 27.7% insecure, and 24.8% unresolved. About 32% of the participants were carriers of the ss genotype; 50% carried the sl genotype and approximately 19% carried the ll genotype.

**Table 1:** Demographic and descriptive data

Characteristic	Value (% or SD)
Age (years, mean‐SD)	19.8 (1.6)
Female (*n*,%)	82 (81.2)
Ethnicity	
European‐American (*n*,%)	34 (33.7)
Asian‐American (*n*,%)	49 (48.5)
Hispanic (*n*, %)	12 (11.9)
African‐American (*n*,%)	2 (2)
Other (%)	4 (4)
BDI (mean‐SD)	6.9 (6.5)
Severe (*n*,%)	1 (0.9)
Moderate (*n*,%)	5 (4.95)
Mild (*n*,%)	10 (9.9)
Minimal (*n*,%)	85 (84.1)
BAI (mean‐SD)	6.1 (5.7)
Severe (*n*,%)	2 (1.9)
Moderate (*n*,%)	4 (3.9)
None or mild (*n*,%)	95 (94.0)
Hollingshead Index (mean‐SD)	47.9 (15.1)
Hollingshead Index groups (*n,*%)	
≥54	44 (43.6)
40–54	32 (31.7)
30–39	9 (8.9)
20–29	9 (8.9)
≤20	7 (6.9)
≥30 Mid/High SES	85 (84.2)
<30 Low SES	16 (15.8)
AAI (*n*,%)	
Secure (“F”)	48 (47.5)
Insecure (“D/E”)	28 (27.7)
Unresolved (“U”)	25 (24.8)
*5‐HTTLPR* genotype (*n*,%)	
S/S	32 (31.7)
S/L	50 (49.5)
L/L	19 (18.8)

AAI, Adult Attachment Interview; BAI, Beck Anxiety Inventory; BDI, Beck Depression Inventory; SES, socioeconomic status.

There were no significant differences in attachment classification on the basis of sex or ethnicity. Neither anxiety nor depression was related to categorical attachment classification (four way [F, D, E, U] or two way [U vs. F]), *SLC6A4*_1pc or *SLC6A4*_2pc methylation, sex or ethnicity, thus neither were included in the final models presented here.

### Continuous attachment outcomes

Hypothesis One. Low SES will be associated with higher unresolved loss & trauma (ULT). Because the direction of the association between methylation and ULT is unclear, we hypothesize that both variables will be associated, but do not state a specific relationship.

Initial linear regression analyses showed that *SLC6A4*_2pc was not significantly associated with UL, UT or ULT. A significant linear relationship was detected between *SLC6A4*_1pc and UL, UT and ULT (*F*_1,98_ = 15.224, *P* < 0.001 *F*_1,98_ = 4.364, *P* = 0.039, *F*_1,98_ = 4.253, *P* = 0.042, respectively). Accordingly, the subsequent analyses focused on *SLC6A4*_1pc. Again, each linear regression model adjusted for ethnicity, sex, and genotype, and included methylation, SES, and their interaction as predictors. SES, methylation and genotype were mean‐centered. Table 2 presents descriptive statistics for variables included in the first set of regressions. The mean for loss alone was 3.23 (SD = 1.8, Range = 1–7) and for trauma alone was 2.3 (SD = 1.8, Range = 1–7). Table 3 presents the results of the regression analyses involving the SES*SLC6A4_1pc methylation. Correlations for the linear analyses are reported in Table A2 in Appendix.

**Table 2:** Descriptive statistics for variables included in the linear regressions examining the associations of SES, *SLC6A4*_1pc methylation, and their interaction

	Mean	Std deviation	*N*
ULT	3.29	1.8	100
UL	2.97	1.8	100
UT	2.3	1.8	40
UT (whole sample)	1.5	1.28	100
SES	0.000	0.36845	100
Genotype	0.000	0.70575	100
Asian‐Amer. versus Others	0.500	0.5025	100
Male versus Female	0.81	0.394	100
*SLC6A4* 1pc1 methylation	0.000	2.01522	100
SES*SLC6A4 1pc1 methylation	−0.104	0.81086	100
UL (UL w/o trauma)	3.33333	1.842446	69
SES	−0.0441	0.32250	69
Genotype	0.1010	0.72702	69
Asian‐Amer. versus Others	0.391	0.4916	69
Male versus Female	0.81	0.394	69
*SLC6A4* 1pc1 methylation	−0.1377	2.09057	69
SES*SLC6A4 1pc1 methylation	−0.1762	0.69666	69

SES, socioeconomic status; UL, unresolved loss; ULT, unresolved loss and trauma.

**Table 3:** Results from the linear regression examining the associations of SES, *SLC6A4*_1pc methylation, and their interaction with four measures unresolved attachment

	*B*	Std. er.	β	*t*	Sig.	CI (95% CI for *B*)	
UL							
SES	0.417	0.496	0.082	0.841	0.403	−0.568	1.401
Asian‐Amer. versus Others	−0.276	0.400	−0.074	−0.689	0.493	−1.070	0.519
Male versus Female	−0.696	0.472	−0.146	−1.476	0.143	−1.633	0.240
Genotype	0.200	0.277	0.075	0.721	0.473	−0.351	0.751
*SLC6A4*_1pc methylation	−0.384	0.096	−0.412	−4.014	0.000	−0.574	−0.194
SES*SLC6A4_1pc methylation	−0.472	0.220	−0.204	−2.146	0.034	−0.909	−0.035
Model: *F*_6,93_ = 4.255, *P* = 0.001, AdR2 = 0.165							
UL w/o Trauma (UT)							
SES	−0.839	0.958	−0.147	−0.876	0.384	−2.755	1.076
Asian‐Amer. versus Others	−0.021	0.498	−0.006	−0.042	0.967	−1.017	0.975
Male versus Female	−0.620	0.575	−0.132	−1.079	0.285	−1.768	0.529
Genotype	0.047	0.325	0.019	0.145	0.885	−0.602	0.697
*SLC6A4*_1pc methylation	−0.434	0.123	−0.492	−3.540	0.001	−0.679	−0.189
SES*SLC6A4_1pc methylation	−1.031	0.427	−0.390	−2.417	0.019	−1.884	−0.178
Model: F_6,62_ = 3.281, *P* = 0.007, AdR2 = 0.17							
UT							
SES	1.521	0.309	0.439	4.919	0.000	0.907	2.136
Asian‐Amer. versus Others	0.140	0.250	0.055	0.560	0.577	−0.356	0.636
Male versus Female	−0.428	0.294	−0.132	−1.453	0.150	−1.012	0.157
Genotype	−0.348	0.173	−0.192	−2.010	0.047	−0.692	−0.004
*SLC6A4*_1pc methylation	0.151	0.060	0.238	2.525	0.013	0.032	0.269
SES*SLC6A4_1pc methylation	0.446	0.137	0.283	3.253	0.002	0.174	0.719
Model: *F*_6,93_ = 7.985, *P* < 0.001, AdR2 = 0.30							
ULT							
SES	1.297	0.520	0.256	2.491	0.015	0.263	2.330
Asian‐Amer. versus Others	−0.200	0.420	−0.054	−0.476	0.635	−1.034	0.634
Male versus Female	−0.845	0.495	−0.178	−1.705	0.091	−1.828	0.139
Genotype	−0.010	0.291	−0.004	−0.033	0.974	−0.588	0.569
*SLC6A4*_1pc methylation	−0.222	0.100	−0.239	−2.207	0.030	−0.421	−0.022
SES*SLC6A4_1pc methylation	−0.029	0.231	−0.012	−0.125	0.901	−0.487	0.430
Model: *F*_6,93_ = 2.226, *P* = 0.047, AdR2 = 0.07							

SES, socioeconomic status; UL, unresolved loss; ULT, unresolved loss and trauma; UT, unresolved trauma.

Results showed that the SES‐*SLC6A4*_1pc methylation interaction was not significantly associated with ULT (*B* = −0.029, *P* = 0.901). Low‐SES was associated with higher ULT (*B* = 1.297, *P* = 0.015). Low methylation was also associated with higher ULT (*B* = −0.222, *P* = 0.030).

Hypothesis Two: SES and methylation will interact such that low‐SES and high methylation will predict higher unresolved trauma (UT).

Results showed a significant SES‐methylation interaction: low‐SES and higher *SLC6A4*_1pc methylation were associated with higher UT (*B* = 0.446, *P* = 0.002). Although main effects should be interpreted with caution in light of the significant interaction, we also detected main effects for SES, genotype, and methylation: low‐SES, increasing counts of the s allele, and higher *SLC6A4*_1pc methylation were each associated with higher UT (*B* = 1.521, *P* < 0.001, *B* = −0.348, *P* = 0.047; *B* = 0.151, *P* = 0.013, respectively).

Hypothesis Three: SES and methylation will interact such that low‐SES and low methylation will predict higher unresolved loss (UL).

Results showed a significant SES‐methylation interaction; low‐SES and low *SLC6A4*_1pc methylation were associated with higher UL (*B* = −0.472, *P* = 0.034). Main effects also showed that lower levels of *SLC6A4_1pc* methylation were associated with higher levels of UL (*B* = −0.384, *P* < 0.001). Results for the remaining variables were insignificant.

As noted above, because we suspected that those participants with UL who also had co‐occurring UT could be pulling methylation levels up, and therefore weakening the SES*SLC6A4_1pc methylation interaction, we ran an analysis eliminating any participant with UT in addition to UL. As expected, low *SLC6A4*_1pc methylation was significantly related to higher UL (*B* = −0.434, *P* = 0.001), and the interaction between SES and methylation was also more robust despite the smaller sample (*B* = −1.031, *P* = 0.019).

Overall, the results of the regressions predicting the continuous measures of attachment showed that a) low‐SES, increasing counts of the s allele, and high methylation, were positively associated with UT and b) low‐SES and low methylation were positively associated with UL, with effects highest when participants with co‐occurring trauma were eliminated from analyses. When UL and UT were collapsed together, however, the methylation*SES interaction was not significant, although main effects were detected for SES and methylation.

Results from the attempt to partially replicate van IJzendoorn et al.'s (2010) genotype‐*SLC6A4* methylation interaction showed that low‐SES and low methylation were associated with higher ULT (*B* = 1.299, *P* = 0.011; *B* = −0.216, *P* = 0.033, respectively), but the genotype*SLC6A4 methylation interaction was insignificant (*B* = 0.134, *P* = 0.283; model; *F*_6,93_ = 2.424, *P* = 0.031 AdR2 = 0.08).

### Categorical classifications

Hypothesis four: AAI categorical classifications (*unresolved* vs. *organized or secure*) will be associated with *SLC6A4* methylation, and we will detect an SES effect within the *unresolved* category, in that low‐SES unresolved participants with have higher methylation than mid/upper‐SES unresolved participants. No differences in methylation will be detected in organized or secure participants across SES.

As noted above, because this analysis includes primarily categorical variables, univariate ANOVA was used to conduct the analyses, and attachment classification was designated the independent variable and methylation the dependent variable. To facilitate comparisons with the analysis using the continuous data, however, we also conducted a regression to determine whether a significant relationship exists between categorical attachment classification as a dependent variable, covarying for SES and *SLC6A4*_2pc methylation as we did in the other models.

*SLC6A4*_1pc methylation and genotype were not associated with categorical attachment whether the analysis involved a four‐way classification (F, D, E, U) or a two‐way classification (organized vs. unresolved). Accordingly, analysis focused on *SLC6A4*_2pc methylation. Because previous studies, as well as the results in the present study, indicate that methylation levels associated with UL run in the opposite direction from methylation associated with UT, and the categorical unresolved classification does not distinguish between those who are unresolved because of loss versus trauma, we did not test for an SES*categorical attachment interaction. Instead, we tested for a SES effect within attachment classifications (unresolved vs. secure, unresolved vs. organized). We suspected that testing for an SES effect within the unresolved classification was more appropriate because the lower SES had higher levels of UT (*F*_1,98_ = 14.729, *P* < 0.001), and theoretically, those living in a lower‐SES environment encounter higher levels of chronic stress. Accordingly, we would expect those participants living in such a high‐risk environment to have higher levels of methylation. Consistent with other studies (Bock 2012), we applied false discovery rate corrections (Benjamini and Hochberg 1995) to our results to control for multiple testing.

Results of the univariate ANOVA with *SLC6A4*_2pc methylation as a dependent variable, and sex, ethnicity, SES and attachment (secure vs. unresolved, organized vs. unresolved) as factors are presented in Table 4. Low‐SES was associated with higher *SLC6A4*_2pc methylation (*B* = 1.270, *P* = 0.023 [0.042 after correction]; *B* = 1.361, *P* = 0.009 [0.023 after correction]), and participants in the unresolved classification had lower levels of methylation than secure or organized participants (*B* = −1.353, *P* = 0.003 (0.010 after correction); *B* = −1.345, *P* = 0.002 (0.009 after correction), respectively).

**Table 4:** Results from analysis of variance of *SLC6A4*_2pc methylation with SES, sex, ethnicity and attachment (U vs. F and Org. vs. U) as factors

Parameter	*B*	Std. error	*t*	Sig.	95% Confidence interval		Partial eta squared
					Lower bound	Upper bound	
AAI: Secure versus Unresolved *n* = 72							
Mid/High SES (0) versus Low (1)	1.270	0.548	2.318	0.023 (0.042)^a^	0.177	2.364	0.074
Male versus Female	0.405	0.521	0.778	0.440 (n.s.)^a^	−0.635	1.445	0.009
Asian‐American versus Others	−0.625	0.422	−1.481	0.143 (n.s.)^a^	−1.467	0.218	0.032
AAI: F versus U	−1.353	0.447	−3.030	0.003 (0.010)^a^	−2.245	−0.462	0.120
AAI: Organized versus Unresolved *n* = 100							
Mid/High SES (0) versus Low (1)	1.361	0.511	2.665	0.009 (0.023)^a^	0.347	2.375	0.070
Male versus Female	0.217	0.456	0.475	0.636 (n.s.)^a^	−0.689	1.123	0.002
Asian‐Amer. versus Others	−0.295	0.356	−0.830	0.409 (n.s.)^a^	−1.001	0.411	0.007
AAI: Org. versus U	−1.345	0.424	−3.170	0.002(0.009)^a^	−2.187	−0.503	0.096

AAI, Adult Attachment Interview; SES, socioeconomic status.

a Corrected for multiple testing using FDR.

Linear regression results showed that a significant relationship between attachment classification (organized vs. unresolved), SES and methylation existed when attachment was made a dependent variable, and SES and methylation were made independent variables. The model was significant (*F*_4,95_ = 4.881, *P* = 0.001 [0.009 after correction]), and main effects existed for SES and methylation; low‐SES was associated with the unresolved classification, and the organized classification had higher levels of methylation (*B* = −0.343, *P* = 0.003 (0.010 after correction); *B* = 0.071, *P* = 0.002 [0.009 after correction]).

**Figure 2:**
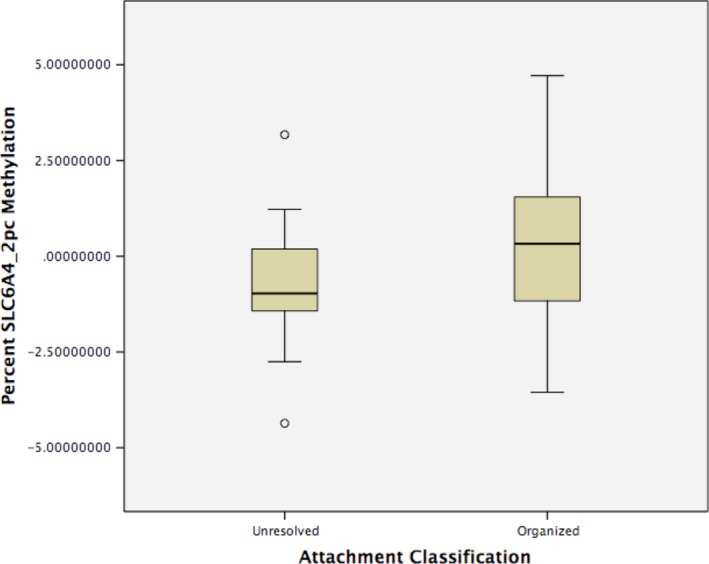
Analysis of variance of *SLC6A4*_2pc methylation with attachment classification (Organized vs. Unresolved) as a factor.

**Figure 3:**
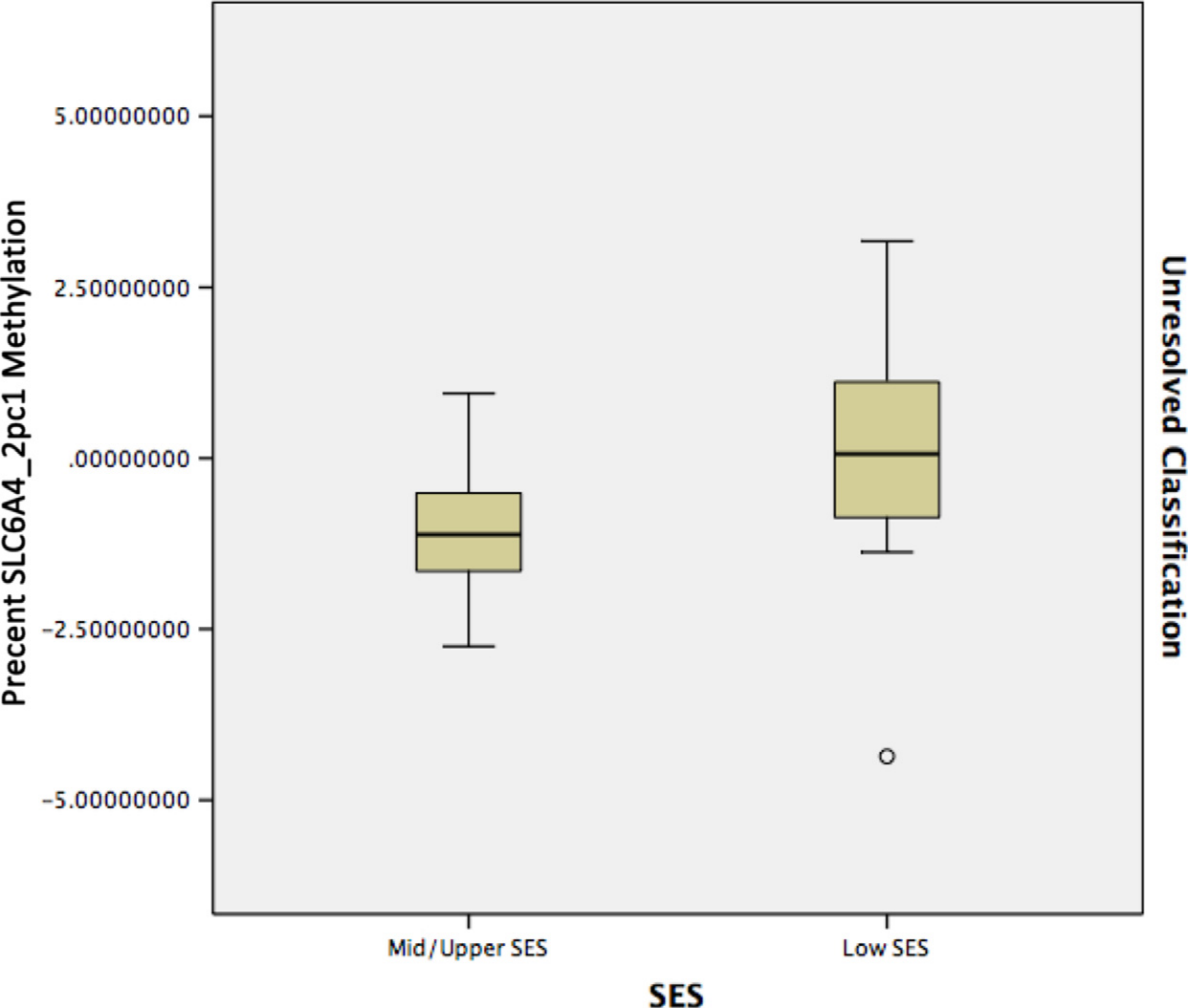
Analysis of variance of *SLC6A4*_2pc methylation with socioeconomic status as a factor among unresolved participants.

After stratifying analyses by attachment classification, we found an SES effect only within the unresolved classification. Low‐SES participants had higher levels of methylation than mid/high SES participants (*B* = −0.983, *P* = 0.012 [0.026 after correction], CI: [−1.733] to [−0.233], PE2 = 0.242) (See Figs. 2 and 3). No significant SES effect in *SLC6A4*_2pc methylation was identified in either the secure or organized classification.

## Discussion

Overall results from this study provide additional evidence that methylation serves as an “interface” between environment and development. Generally, we found that *SLC6A4* methylation, SES, and unresolved attachment classifications were associated. Specifically, results showed that the SES*SLC6A4 methylation and genotype*SLC6A4 methylation interactions were not associated with ULT, although we did find main effects for SES and methylation in both models. Interactions between SES and *SLC6A4* methylation were, however, significant, and had unique patterns of effects, when trauma and loss were separated. Low‐SES and higher *SLC6A4* methylation were associated with higher UT. Low‐SES and lower *SLC6A4* methylation were associated with higher levels of UL. Results were most robust when loss without trauma was used as the dependent variable giving support to the suggestion that *SLC6A4* methylation levels for UL and UT do run in the opposite directions.

Our findings using AAI categorical data were consistent with those from the continuous analysis, increasing confidence in the patterns of association; across SES, the unresolved category had lower levels of *SLC6A4* methylation than the organized or secure classification. We also found an SES effect within the unresolved categorical classification: Those participants falling into the low‐SES unresolved category had higher levels of *SLC6A4* methylation than the mid/high SES unresolved individuals. There were no differences detected between individuals within the secure or organized classifications.

We were not able to replicate findings from van IJzendoorn et al. (2010) with respect to ULT. As noted above, the most likely reason for the different findings is that their sample consisted of middle class, adopted (at birth or close to birth), primarily Caucasian adults that may have had lower levels of trauma than the present sample. Thus, the differing methylation levels associated with UT (or low‐SES) and loss did not cancel each other out, as they appeared to in this sample. Note that this study never specifically targeted a traumatized population for enrollment. In fact, the level of trauma found within this relatively high functioning college sample was surprising. The number of participants with UT was low (*n* = 40), especially when UT scores were averaged with all those participants showing no signs of trauma. Nevertheless, one of the messages of this study is that it is possible that even low numbers of participants with UT, especially when combined with a low‐SES sample, may impact results. Accordingly, we recommend that future methylation analyses keep UT and UL separated until these questions are examined within a larger sample of individuals reporting trauma.

### Effects of SES

SES played an important role in these analyses. For example, within the unresolved classification, low‐SES participants had higher levels of methylation than mid/upper SES participants, whereas SES had no effect within the secure or organized classification. We also found that a SES*methylation interaction was associated with higher UL and UT. These findings are at least consistent with the hypothesis that (1) SES is a critical variable that needs to be taken into account in epigenetic studies, and (2) security of attachment may impact epigenetic processes, and may buffer the impact of low‐SES on epigenetic processes such as methylation. At a minimum, these findings also support further research in this area.

Why would low‐SES unresolved individuals have higher methylation levels than mid/upper‐SES unresolved individuals? The most obvious explanation is that low‐SES participants had significantly higher mean levels of UT than mid/upper‐SES participants. This study, consistent with previous research (Beach et al. 2010), has found that higher levels of *SLC6A4* methylation are associated with trauma. It is also possible that some low‐SES participants may be dealing with two major sources of trauma; child abuse and poverty. There is growing evidence that low‐SES impacts methylation (Borghol et al. 2011; Beach et al. 2014a,b). Moreover, when individuals are raised in a low‐SES environment, even if they enter the middle class in adulthood, the epigenetic “residue” or “scar” of childhood poverty can still be identified (Miller et al. 2009; Borghol et al. 2011). The participants here were just out of childhood. It would hardly be surprising, therefore, that the effects of exposure to a low‐SES environment might be detectable in this sample.

How might security of attachment influence the impact of low‐SES on methylation levels? The answer to this question requires consideration of two points. First, methylation may be much like cortisol in that too much or too little is unassociated with optimal health. For example, recent research found that the number of traumatic events experienced was positively associated with the risk for post‐traumatic stress disorder (PTSD) in individuals with low *SLC6A4* methylation (Koenen et al. 2011). On the other hand, higher levels of *SLC6A4* methylation have been found to be associated with trauma (Beach et al. 2010, 2011). In other words, those with low levels of methylation might be particularly sensitive to environmental events, while those with high levels of methylation may be biologically coping with extreme environmental experiences such as trauma. Although it may be adaptive to effectively shut down a gene for environmental sensitivity in the face of abuse, individuals may pay a price for high methylation levels. For example, greater methylation levels of the oxytocin gene has been linked to conduct disorder (Dadds et al. 2014), and it may be that the decrease in environmental sensitivity associated with high methylation levels found here potentially explain some of the resistance to intervention associated with diagnoses such as conduct disorder. Accordingly, it could be that like cortisol, balance in methylation levels is associated with optimal health.

Second, it is thought that stress is one of the mechanisms through which adverse life experiences such as poverty are associated with disease; stress triggers sympathetic nervous system activation, which leads to increased production of neurotransmitters like noradrenaline, which can then induce proinflammatory gene expression (Eisenberger and Cole 2012). Epigenetic processes that control gene expression may be one intermediary link between low‐SES, stress, and subsequent poor health.

Accordingly, how does security of attachment influence the impact of low‐SES on methylation levels? Recall that the attachment relationship is thought to create or moderate the child's ability to regulate stress with secure children developing optimal self‐regulation (Loman and Gunnar 2010). Ultimately, the essence of attachment security is balance. As Ainsworth pointed out long ago, babies classified as secure are able to demonstrate balance between exploring new environments and attachment to parents (Ainsworth et al. 1978). In the AAI, “An individual high in attachment security is able to discuss experiences with parents with balance and a sense of perspective, without either cutting off or being overwhelmed when asked to talk about attachment experiences” (Wampler et al. 2003, p. 498). A secure individual is able to confront stressful events while using relationships with sensitive and responsive attachment figures to maintain emotional balance. For example, a child living in poverty might be very well aware of the dangers inherent in his environment but still feel safe knowing that a parent figure is a reliable, appropriate, and predictable source of protection. Attachment security would not diminish the child's perceptions of the dangers of living in poverty, but add to those perceptions the knowledge that when real danger arises, he or she has a reliable source of safety. If anything, security may permit the child to be more fully aware of threat, an awareness that is tolerable precisely because the child does have a source of safety upon which to rely. Previous studies using “Stroop” executive function tasks have found that individuals classified as securely attached show slow response latencies (i.e., use more time to process threatening information) and remember more words (even threatening words) than individuals classified as insecure (Zeijlmans van Emmichoven et al. 2003). Accordingly, stress signaling is controlled when a realistic balance is maintained between awareness of threat and a sense of safety. Under these conditions, even a child living in poverty that is carrying the s allele and possesses low methylation levels might avoid negative health outcomes such as depression, anxiety, or other stress‐triggered inflammatory diseases. Indeed, environmental sensitivity may benefit a securely attached child living in poverty since the child has a greater need to reap the benefits of a secure attachment relationship. In related literature, Chen et al. (2011) found that adults who were raised in a low‐SES home but who reported high levels of “maternal warmth” exhibited lower levels of proinflammatory signaling. More generally, in addition to Puig et al.' (2013) finding of higher numbers of inflammatory diseases in insecure compared to secure participants, Gouin et al. (2009) found that among married couples, spouses classified as insecure‐avoidant showed higher levels of IL‐6, a proinflammatory cytokine, during marital conflict. Research into the association between attachment and health is just beginning and we hope to examine this issue in future studies.

### Questions raised

There are some questions that this cross‐sectional study cannot answer with certainty. For example, it is possible that this study reveals two processes that are associated with unresolved attachment. Two studies have now found that *SLC6A4* methylation is associated with unresolved attachment. Genotype is inherited; it may be that lower methylation levels could also be inherited. High *SLC6A4* methylation, however, appears to be associated with environmental experiences, namely abuse. It would be adaptive for an organism to turn off an environmentally sensitive gene in the face of abuse, particularly if it is chronic. Whether low levels of methylation are an inherited biological vulnerability while high levels reflect a reaction to environmental experiences can only be answered with longitudinal studies.

### Strengths and limitations

The primary strength of the study was the use of what are considered “gold standard” measures such as the AAI and the Hollingshead. Second, this study used blood instead of buccal swabs to obtain DNA, a more difficult process but one that is thought to involve less risk of contamination. Third, our sample size appears to be roughly similar to other studies that focus on methylation and social experience, even when the genotype is partial focus of the analysis (van IJzendoorn et al. 2010; Vijayendran et al. 2012; Reiner et al. 2015), suggesting that we have reasonable power for these models. Methylation studies generally require much lower sample sizes than genotype studies because methylation is a continuous variable. Fourth, results were robust to adjustments for multiple testing.

There are a number of limitations in this study. First, although our sample size was acceptable for a methylation study, especially one using gold standard measures of attachment, a larger sample size might strengthen confidence in the findings and allow for the exploration or racial and sex differences in effects. Accordingly, we intend to expand the sample size and the number of measures used in the future. Second, because the study did not specifically target a traumatized population, the number of participants with actual trauma experiences (*n* = 40) was low, and subsequently the level of UT in the entire sample was low. Accordingly, our findings with respect to trauma should be viewed with caution, although as noted, other studies with larger samples of trauma have similar findings with respect to *SLC6A4* methylation. Future studies should, nevertheless, enroll a larger sample size targeting participants with a broader range of trauma exposure. Third, findings from our sample of college students in late adolescence must be generalized to other ages with caution. Fourth, as stated earlier, our cross‐sectional study limits our ability to discern when methylation profiles are a function of inheritance, and thus act as a source of biological vulnerability, and when such profiles are a reaction to experience. Fifth, although blood is considered the optimal peripheral source for methylation data, whole blood also contains a number of different cell types, a phenomenon known as cellular heterogeneity. The cellular heterogeneity of blood can certainly lead to cell‐composition effects influencing our analyses: This variability also occurs in the context of gender, age, and race (Ji et al. 2010; Zhang et al. 2011; Peters et al. 2015). It does appear, however, that for many loci, there is stability in methylation across time and tissue type (Talens et al., 2010). More research is needed to clarify what role cellular heterogeneity plays in methylation studies conducted with blood. Finally, gene expression data was not available in these data, and future work examining these associations with gene expression may be informative.

### Conclusion and future directions

The findings of this study add to the literature exploring the association between SES and health. In the context of the known associations between security of attachment and lower rates of inflammatory disease or responses in adulthood (Gouin et al. 2009; Puig et al. 2013), as well as associations between parenting behaviors such as maternal warmth and epigenetic processes (Chen et al. 2011), the present study furthers research by suggesting that security of attachment may act as a protective factor against the impact of low‐SES on methylation. Longitudinal research confirming these findings is needed to ascertain whether attachment security safeguards health through regulation of stress‐responsive genes. In light of the significant sample size required in epigenetic studies and the cost and complexity involved in the gold standard measure of attachment used here, collaborations will enhance the feasibility of future studies. Our findings also reveal that it is critical to control for SES and consider it as a moderator of effects, which should be easy for most studies to examine. Given our finding that attachment classification relates to the epigenetic regulation of stress, it is plausible that such relationships may be relevant to other related biological phenomenon such as immune function. Epigenome wide association (EGWA) studies that can assess the broader biological impact of attachment relationships are likely to be informative. EGWA studies have recently discovered interactions between SES and environmental experience on health (Uddin et al. 2013). The results reported here reinforce the notion that relational, biological and socioeconomic factors relate to each other in a dynamic process—understanding these complexities is a worthwhile endeavor that may inform prevention and intervention efforts related to early life social relationships.

## Conflict of Interest

None declared.

## Appendix

**Table A1:** Depression (BDI), anxiety (BAI), ethnicity, sex and methylation and attachment

A. We note that all analyses contain a variable labeled Asian American versus others. The term “others” refers to all non‐Asian‐American participants. This variable was added because the only differences detected in genotype or in *SLC6A4*_1pc methylation between ethnic groups was between Asian Americans and Euro‐Americans (χ^2^ = 22.606, *P* < 0.001, *F *=* *8.031, *P *=* *0.006, respectively). We added all remaining ethnic groups to the “other” category out of an abundance of caution. Although this is primarily a female study, some differences in *SLC6A4*_1pc methylation by sex were detected (*F*_1,98_ = 11.686, *P* = 0.001). Accordingly all *SLC6A4*_1pc methylation analyses added sex to the analysis. Although no differences in sex or ethnicity were detected in *SLC6A4*_2pc methylation we controlled for both variables, as well.
B. Neither anxiety nor depression was related to categorical attachment classification (four way [F, D, E, U] or two way [U vs. F]), *SLC6A4*_1pc or *SLC6A4*_2pc methylation, sex or ethnicity. We initially detected a significant difference in depression between organized versus unresolved participants (*F*_1,99_ = 4.470), *P* = 0.037), but significance disappeared once we controlled for SES. Low‐SES participants did have higher levels of depression than mid/upper SES participants (*F*_1,98_ = 12.90, *P* = 0.001) but it should be remembered that overall this was not a highly depressed sample (93% scored as having no or mild depression). Nevertheless, out of an abundance of caution, we ran the univariate ANOVA models with depression and found depression insignificant.
C. Continuous UL, trauma and loss or trauma was unrelated to anxiety. UL (w/o trauma) (*F*_1,78_ = 0.667, *P* = 0.417) and UT (*F*_1,99_ = 0.713, *P* = 0.749) was unassociated with depression. UL (including those participants with loss and trauma) and UL and trauma was initially associated with depression (*F*_1,97_ = 5.824, *P* = 0.02); *F*_1,99_ = 6.305, *P* = 0.014 respectively), but after SES was controlled for or added to the model significance disappeared. Moreover, the association between depression and UL, and loss or trauma, was due to one outlier. Removing that subject eliminated the association. In addition, we ran the models (UL and loss and trauma) with depression and found depression was insignificant. Accordingly, depression was removed from all analyses.

BAI, Beck Anxiety Inventory; BDI, Beck Depression Inventory; SES, socioeconomic status; UL, unresolved loss; UT, unresolved trauma.

**Table A2:** Correlations between UL, UL (w/o trauma), UT and ULT and SES, sex, ethnicity, genotype, *SLC6A4_1pc*, methylation and SES*SLC6A4 methylation interaction

	U score	SES	Male versus Female	Ethnic.	Geno.	*SLC6A4*_*1pc* meth.	SES**SLC6A4*_*1pc* meth.
UL (*n* = 100)							
UL	1.000	0.146	0.006	0.032	0.043	−0.367^a^	−0.257^a^
SES	0.146	1.000	0.142	0.109	−0.191^a^	−0.142	−0.239^a^
Male versus Female	0.006	0.142	1.000	−0.025	−0.090	−0.326^a^	−0.053
Ethnicity	0.032	0.109	−0.025	1.000	−0.441^a^	−0.283^a^	0.101
Genotype	0.043	−0.191^a^	−0.090	−0.441^a^	1.000	0.146	0.012
*SLC6A4*_*1pc* meth.	−0.367^a^	−0.142	−0.326^a^	−0.283^a^	0.146	1.000	0.110
SES**SLC6A4*_*1pc* meth.	−0.257^a^	−0.239^a^	−0.053	0.101	0.012	0.110	1.000
UL (w/o trauma) (*n* = 69)							
UL (UL w/o UT)	1.000	0.219^a^	0.007	0.154	−0.064	−0.361^a^	−0.252^a^
SES	0.219^a^	1.000	0.174	0.081	−0.174	−0.274^a^	−0.660^a^
Male versus Female	0.007	0.174	1.000	0.007	−0.173	−0.375^a^	0.042
Ethnicity	0.154	0.081	0.007	1.000	−0.462^a^	−0.316^a^	−0.066
Genotype	−0.064	−0.174	−0.173	−0.462^a^	1.000	0.202^a^	0.088
*SLC6A4*_*1pc* meth.	−0.361^a^	−0.274^a^	−0.375^a^	−0.316^a^	0.202^a^	1.000	−0.091
*SES***SLC6A4*_*1pc* meth.	−0.252^a^	−0.660^a^	0.042	−0.066	0.088	−0.091	1.000
UT (*n* = 100)							
UT (*n* = 100)	1.000	0.361^a^	−0.147	0.110	−0.251^a^	0.206^a^	0.207^a^
SES	0.361^a^	1.000	0.142	0.109	−0.191^a^	−0.142	−0.239^a^
Male versus Female	−0.147	0.142	1.000	−0.025	−0.090	−0.326^a^	−0.053
Ethnicity	0.110	0.109	−0.025	1.000	−0.441^a^	−0.283^a^	−0.049
Genotype	−0.251^a^	−0.191^a^	−0.090	−0.441^a^	1.000	0.146	0.012
*SLC6A4*_*1pc* meth.	0.206^a^	−0.142	−0.326^a^	−0.283^a^	0.146	1.000	0.110
*SES***SLC6A4*_*1pc* meth.	0.207^a^	−0.239^a^	−0.053	−0.049	0.012	0.110	1.000
UL/trauma (*n* = 100)							
UL/trauma (UTL)	1.000	0.262^a^	−0.062	0.048	−0.048	−0.204^a^	−0.088
SES	0.262^a^	1.000	0.142	0.109	−0.191^a^	−0.142	−0.239^a^
Male versus Female	−0.062	0.142	1.000	−0.025	−0.090	−0.326^a^	−0.053
Ethnicity	0.048	0.109	−0.025	1.000	−0.441^a^	−0.283^a^	−0.049
Genotype	−0.048	−0.191^a^	−0.090	−0.441^a^	1.000	0.146	0.012
*SLC6A4*_*1pc* meth.	−0.204^a^	−0.142	−0.326^a^	−0.283^a^	0.146	1.000	0.110
*SES***SLC6A4*_*1pc* meth.	−0.088	−0.239^a^	−0.053	−0.049	0.012	0.110	1.000

SES, socioeconomic status; UL, unresolved loss; ULT, unresolved loss and trauma; UT, unresolved trauma.

a *P* < 0.05.
